# Characterization of Primary Cilia in Normal Fallopian Tube Epithelium and Serous Tubal Intraepithelial Carcinoma

**DOI:** 10.1097/IGC.0000000000001321

**Published:** 2018-08-08

**Authors:** Zakia A. Abdelhamed, Thomas A. Ryan, Martin Fuller, Camilla Coulson-Gilmer, Dina I. Abdelmottaleb, Tian-Li Wang, Jen-Chun Kaun, Peiyi Wang, Richard Hutson, Nafisa Wilkinson, Sandra M. Bell, Colin A. Johnson

**Affiliations:** * Leeds Institute of Biomedical and Clinical Sciences, University of Leeds, Leeds, United Kingdom;; † Department of Anatomy and Embryology, Faculty of Medicine, Al-Azhar University, Cairo, Egypt;; ‡ Astbury Centre for Structural Molecular Biology, University of Leeds, Leeds, United Kingdom;; § Department of Zoology, Faculty of Science, Benha University, Benha, Egypt;; ∥ Departments of Pathology, Oncology, and Gynecology/Obstetrics, The Sidney Kimmel Comprehensive Cancer Center, School of Medicine, Johns Hopkins University, Baltimore, MD;; ¶ Institute of Oncology, and; # Department of Histopathology, Leeds Teaching Hospitals NHS Trust, Leeds, United Kingdom.

**Keywords:** Fallopian tube, Primary cilia, Sonic Hedgehog signaling, Smoothened, Serous tubal intraepithelial carcinoma, Dynein axonemal heavy chain 5

## Abstract

**Objectives:**

The aim of this study was to investigate the distribution of primary cilia on secretory cells in normal fallopian tube (FT) and serous tubal intraepithelial carcinoma (STIC).

**Methods:**

Fallopian tube tissue samples were obtained from 4 females undergoing prophylactic hysterectomies and 6 patients diagnosed with STIC. A *mogp*-TAg transgenic mouse STIC sample was also compared with a wild-type mouse FT sample. Serous tubal intraepithelial carcinoma was identified by hematoxylin and eosin staining and confirmed by positive Ki-67 and p53 immunohistochemical staining of tissue sections. We assessed the relative distribution of primary cilia on secretory cells and motile cilia on multiple ciliated cells by immunofluorescence and immunohistochemical staining. Ciliary function was assessed by immunofluorescence staining of specific ciliary marker proteins and responsiveness to Sonic Hedgehog signaling.

**Results:**

Primary cilia are widespread on secretory cells in the ampulla, isthmus, and in particular, the fimbriae of human FT where they may appear to mediate ciliary-mediated Sonic Hedgehog signaling. A statistically significant reduction in the number of primary cilia on secretory cells was observed in human STIC samples compared with normal controls (*P* < 0.0002, Student *t* test), supported by similar findings in a mouse STIC sample. Immunohistochemical staining for dynein axonemal heavy chain 5 discriminated multiple motile cilia from primary cilia in human FT.

**Conclusions:**

Primary cilia are widespread on secretory cells in the ampulla, isthmus, and in particular, the fimbriae of the human FT but are significantly reduced in both human and mouse STIC samples. Immunohistochemical staining for ciliary proteins may have clinical utility for early detection of STIC.

## Abstract 2

Supplemental digital content is available in the text.

Human fallopian tubes (FTs) are tubular seromucosal organs that connect the ovaries to the uterus in females. The anatomy of the FT comprises distinct segments (lateral to medial) as follows: the infundibulum and associated fimbriae near the ovary, the ampulla forming the major portion of the lateral FT, and the isthmus that links to the uterus.^1^ In cross-section, the FT has distinct histological layers, comprising of the serosa, smooth muscle, subserosa, lamina propria, and mucosal layers. The innermost layer of normal FT, the mucosa, contains columnar epithelium that consists of 2 main types of mucosal cell, secretory, and ciliated cells. The secretory cells, also known as peg cells, are generally considered to be nonciliated, to contain apical granules, and to produce tubular fluid. This contains nutrients for spermatozoa and oocytes, as well as promoting capacitation of sperm before oocyte fertilization.^2^

Ciliated cells occur throughout the FT but predominant at the apex of mucosal folds. Ciliated cells carry multiple motile cilia (MCCs). The coordinated beating of MCCs is thought to provide the fluid flow needed to move an oocyte through the FT to the uterus. Multiple motile cilia have a canonical “9 + 2” ultrastructure of microtubules in the ciliary axoneme, consisting of 9 peripheral doublet microtubules surrounding 2 single central microtubules. The “9 + 2” axonemal ultrastructure is also observed in other motile cilia of the respiratory tract and sperm flagella.

In contrast to MCCs, primary cilia have a “9 + 0” axonemal ultrastructure and are ubiquitous sensory organelles. They are displayed as single protrusions on the surface of most vertebrate cells and are essential for mechano-, chemo-, and photosensation in many mammalian cell types during the G_1_/G_0_ phases of the cell cycle.^3^,^4^ Primary cilia have a complex ultrastructure with compartmentalization of molecular components that combine in functional modules. The loss or mutation of these components can disrupt ciliary functions such as the control of protein entry and exit from the cilium, regulation of signaling cascades, and control of the cell cycle.^5^ These signaling pathways include the Hedgehog, Wnt, and Notch signaling pathways^6^–^9^ and a common mechanism for regulating these pathways appears to be the discrete compartmentalization of signaling components to the cilium. As a paradigm for other pathways, Smoothened (Smo), the coreceptor and transducer for Sonic Hedgehog (Shh), translocates into and then activates Gli transcription factors within the cilium.^10^ Canonical Wnt/β-catenin signaling is also constrained by compartmentalization of the Wnt signaling component Jouberin and β-catenin away from the nucleus and into the cilium.^11^ More recently, the mTOR,^12^,^13^ Hippo,^14^,^15^ and TGFβ^16^ signaling pathways have all been shown to be regulated through ciliary-dependent mechanisms, with diverse consequences on cell proliferation and size, differentiation, autophagy, apoptosis, and tumorigenesis. Loss of regulated cell growth is a hallmark of cancer, and loss of primary cilia has been associated with tumorigenesis in many cancers including breast, melanoma, pancreatic, prostrate, and ovarian.^17^–^21^

Recent advances in understanding the causes of ovarian cancer suggest that many high-grade serous cancers (HGSCs) originate from the epithelium of the FT.^22^–^24^ Identification of HGSC in *BRCA1* and *BRCA2* carriers led to risk-reducing bilateral salpingo-oophorectomy and very careful pathological examination of the FT that identified “serous tubal intraepithelial carcinoma” (STIC) as the early-stage precursor of these cancers.^22^ Newly developed genetically engineered mouse models for ovarian cancer have confirmed the site of origin in the FT and subsequent progression from STIC to HGSC. For example, the *mogp*-TAg transgenic mouse expresses the SV40 large T-antigen (TAg) under the control of the mouse müllerian-specific *Ovgp-1* promoter.^25^ TAg binds and inactivates p53 in the FT causing a progression from normal tubal epithelium to noninvasive precursor lesions resembling human “p53 signatures” and STIC, with development of invasive HGSC in the ovary.^26^ However, little is currently known about the molecular events leading to the development of STIC.

Primary cilia in the mammalian oviduct have been described previously on secretory cells,^27^,^28^ but their functional role is unknown. The accepted view in the literature is that the human FT ciliated epithelium predominantly displays only MCCs.^29^ A more recent study analyzed the motor protein composition of MCCs in the human FT,^30^ but did not assess the presence, composition, or function of primary cilia in this tissue. To address these issues, the present study has visualized functional primary cilia in secretory cells of the human FT and has assessed primary cilia incidence on secretory cells in STIC samples from both human patients and a mouse model.

## MATERIALS AND METHODS

### Subjects, Ethical Approval, and Tissue Specimens

Normal, undiseased specimens of fresh FT tissue were obtained from 4 females who underwent prophylactic hysterectomies. Six patients with STIC were identified from formaldehyde-fixed paraffin embedded (FFPE) hematoxylin and eosin (H&E) sections by the reporting histopathologist (N.W.), with confirmatory immunohistochemistry (IHC) performed for p53 and Ki-67. Tissues samples were provided by the Leeds Multidisciplinary Research Tissue Bank with approval from the Leeds East Research Ethics Committee (10/H1306/7). Written informed consent to participate in the study was obtained from each participant. Serous tubal intraepithelial carcinoma tissue samples were obtained from the *mogp*-TAg transgenic mouse, which develops ovarian HGSC originating from the FT and a wild-type mouse control FT sample, as described previously.^25^,^26^

### Primary Cell Culture

Fresh human FT samples were collected immediately after surgery and placed on ice in PBS containing 1% penicillin/streptomycin (P/S) and then washed 3 times in PBS/1% P/S to eliminate contamination. The fimbriae tips and mucosal layer of cells were removed by gently scraping a scalpel along the internal FT surface. The whole sample was then incubated in 10-mL trypsin for 20 minutes at 37°C. Then, 5-mL fetal calf serum and 5-mL RPMI medium supplemented with 10% fetal calf serum and 1% P/S were added. Next, the cell suspension was removed and centrifuged for 5 minutes at 200*g*. A second trypsinization step was performed, as described previously, on the remaining tissue fragments and then the 2 cell supernatants combined, centrifuged resuspended in 2-mL media and transferred to a tissue culture plate. After allowing 48 hours for the epithelial FT cells to attach, the medium was changed and contaminating fibroblasts removed by fractional trypsinization if required. For measurement of Shh signaling pathway activity, primary cells at 80% confluency were treated for 4 hours with 1 μg/mL recombinant human Shh protein (R&D Systems, Inc, Abingdon, United Kingdom), 2 μM SAG (Enzo Life Sciences Ltd, Exeter, United Kingdom), or vehicle negative control (0.1% DMSO).

### Antibodies

The following primary antibodies were used: mouse monoclonal anti–polyglutamylated tubulin (1:1000; clone GT335; cat. no. ALX-804-885; Enzo Life Sciences Ltd); mouse monoclonal anti–acetylated-α-tubulin (1:1500; clone 6-11B-1; Sigma-Aldrich Co LLC, St Louis, Mo); rabbit polyclonal anti–γ-tubulin (1:1000; Abcam Ltd, Cambridge, United Kingdom); affinity purified rabbit polyclonal anti–adenylate cyclase III (ADCY3; 1:500; cat. no. RPCA-ACIII; EnCor Biotechnology Inc, Gainsville, Fla); affinity purified rabbit polyclonal anti-INPP5E (1:200; cat. no. 17797-1-AP; Proteintech Inc, Rosemont, Ill); affinity purified rabbit polyclonal anti-ARL13B (ARL2L1) (1:1000; cat. no. 17711-1-AP; Proteintech Inc); affinity purified rabbit polyclonal anti-IFT88 (1:500; cat. no. 13967-1-AP; Proteintech Inc); rabbit polyclonal anti-Smoothened (1:200; cat. no. bs-2801R; Bioss Inc, Woburn, Mass); mouse monoclonal anti–Ki-67 clone MIB-1 (1:100; cat. no. M7240; Dako UK Ltd, Ely, Cambridgeshire, United Kingdom); mouse monoclonal p53 (1:100; cat. no. NCL-L-p53-Do7; Leica Biosystems Inc, Buffalo Grove, Ill); and rabbit polyclonal anti–dynein axonemal heavy chain 5 (DNAH5; 1:800; cat. no. HPA037470; Sigma-Aldrich Co). Secondary antibodies were Alexa-Fluor568 or 488-conjugated goat anti–mouse IgG and goat anti–rabbit IgG (Thermo Fisher Scientific Inc).

### Preparation of Tissue Sections, Histology, and Immunohistochemistry

Fallopian tube tissue samples were dissected and fixed in 4% para-formaldehyde and embedded in paraffin wax. Thin sections (4 μm) were deparaffinized and rehydrated by standard methods. Sections were stained with H&E (VWR International Ltd) for 2 minutes, then dehydrated in ethanol, cleared in xylene, and mounted in DPX. For IHC, tissue sections were deparaffinized and rehydrated. Epitope recovery was obtained by boiling in 1 mM EDTA pH8.0, for 2 minutes using pressure cooker, followed by 20 minutes cooling. Blocking and application of primary antibodies was as described.^31^ Appropriate HRP-conjugated secondary antibodies (Dako UK Ltd) were used (final dilutions of ×10,000–25,000). Sections were developed in “Sigma Fast” 3,3′-diaminobenzidine (DAB) with CoCl_2_ enhancer and counterstained with Mayer's hematoxylin (Sigma-Aldrich Co). For dual-color IHC staining, an “ImmPRESS Duet” double staining HRP/AP polymer kit (Vector Labs, United Kingdom) was used. This visualized anti**–**rabbit IgG-AP as red and anti**–**mouse IgG-HRP as brown staining.

### Immunofluorescence and Confocal Microscopy

Primary cells were seeded at 1.5 × 10^5^ cells/well on glass coverslips in 6-well plates and fixed in ice-cold methanol (5 minutes at −20°C) or 2% paraformaldehyde (20 minutes at room temperature). Permeabilization, blocking methods, and immunofluorescence (IF) staining were essentially as described previously.^31^ Primary antibodies were used at final dilutions of ×200 to 1000. Secondary antibodies were diluted ×500. Tissues were permeabilized and blocked (0.1% Triton-X 100 with 10% normal goat serum in PBS) for 30 minutes at room temperature and then incubated in primary antibodies overnight at 4°C. After several PBS washes, they were incubated with secondary antibodies for 30 minutes at room temperature. Samples were mounted on glass slides using Vectashield with 4′,6-diamidino-2-phenylindole (DAPI; Vector Laboratories Ltd, Peterborough, United Kingdom). Imaging was carried out using a Nikon Eclipse TE2000-E system, controlled and processed by EZ-C1 3.50 (Nikon UK Ltd, Kingston-upon-Thames, United Kingdom) software.

### Scanning and Transmission Electron Microscopy

For scanning electron microscopy, fresh FT samples were fixed in 0.4% para-formaldehyde for 30 minutes at room temperature and imaged using a Hitachi S-3400 N scanning electron microscope. For TEM, fresh FT samples were fixed with 2.5% glutaraldehyde in 0.1-M phosphate buffer for 2 hours and washed twice for 30 minutes in 0.1-M phosphate buffer. Samples were post-fixed in 1.0% osmium tetroxide in 0.1-M phosphate buffer, washed twice as above, and dehydrated using an ascending alcohol series. Samples were then washed twice with propylene oxide for 20 minutes each, and embedded in an increasing series of “Araldite” epoxy resin: propylene oxide ratio (50:50, 75:25, and 100:0; 3–8 hours for each change). Samples were allowed to polymerize overnight at 60°C. Semi-thin sections (0.5–2.0 μm) were cut and stained with 1.0% toluidine blue in 1.0% sodium borate to aid with orientation. Ultrathin (80–100 nm) sections were placed on 3.05-mm copper grids, stained with saturated uranyl acetate for 30 minutes, and Reynold's lead citrate for 5 minutes and imaged using a JEOL-JEM1400 microscope with direct magnification at ×10,000.

### Primary Cell Transfection and Reporter Assays

For measurement of Shh signaling pathway activity, primary cells at 80% confluency were transfected using Lipofectamine 2000 (Thermo Fisher Scientific Inc) according to the manufacturer’s instructions and as described previously.^32^ Sonic Hedgehog pathway activation was as described previously, and activity was measured using the “Cignal” GLI dual luciferase reporter assay (QIAGEN GmbH, Hilden, Germany). Luciferase activity was assayed with the Dual-Luciferase Reporter Assay system (Promega Corp, Madison, Wis) on a Mithras LB940 luminometer (Berthold Technologies GmbH & Co KG, Bad Wildbad, Germany). Results reported are from 3 independent biological replicates. Normal distribution of data was confirmed using the Kolmogorov-Smirnov test (“Prism,” GraphPad Software Inc, La Jolla, Calif). Pairwise comparisons were analyzed with Student 2-tailed *t* test using InStat (GraphPad “Prism”).

## RESULTS

### Regional Distribution of Primary Cilia in Human FTs

**FIGURE 1:**
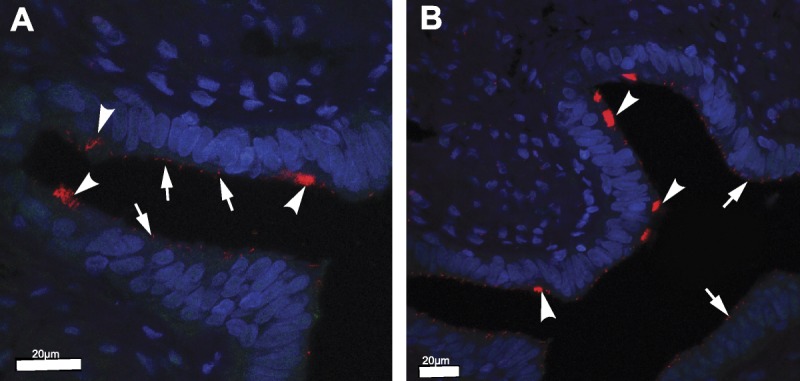
Immunofluorescence staining of longitudinal sections from the fimbrial end of normal human FT samples. Ciliated cells containing MCC (arrows) and secretory cells containing primary cilia (arrowheads) were identified using polyglutamylated tubulin (red; a marker of ciliary axonemes in both MCCs and primary cilia). Nuclei were counterstained with DAPI (blue). Scale bars = 20 μm.

**FIGURE 2:**
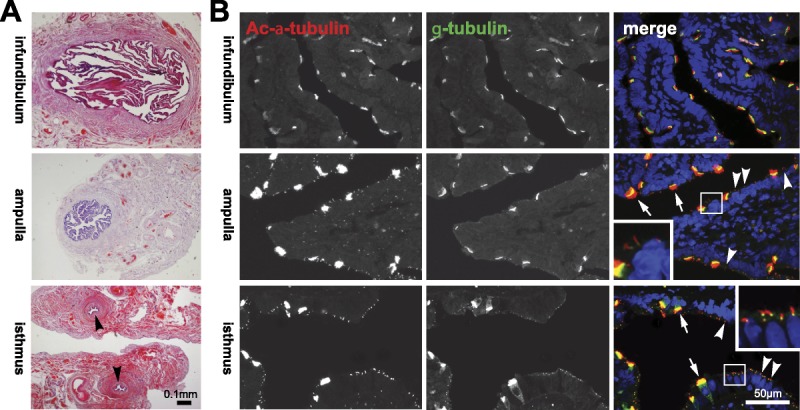
Regional distribution of MCC and primary cilia in the human FT. A, Transverse sections of the FT at the level of the infundibulum, ampulla, and isthmus (indicated by arrowheads), counterstained with H&E. Scale bar = 0.1 mm. B, FT sections at the indicated anatomical regions IF stained for acetylated (Ac) α-tubulin (red; a marker of ciliary axonemes in both MCCs and primary cilia) and γ-tubulin (green; a marker of the ciliary basal body in both cilia types). Nuclei were counterstained with DAPI (blue). Merged color channels are shown on the right. Arrows indicate MCCs and arrowheads indicate primary cilia, with detail shown in magnified insets. Single immotile primary cilia are displayed on secretory peg cells. Scale bar = 50 μm.

Previous studies have reported MCC in human FT samples. To investigate if single primary cilia were also present, we examined FFPE human FT samples from prophylactic hysterectomies by IF staining and confocal microscopy. Initially, we investigated the fimbrial end in longitudinal section of the FT. We stained for polyglutamylated tubulin (a marker of ciliary axonemes in both MCCs and primary cilia). Multiple motile cilia were predominantly distributed at the apex of mucosal folds in the fimbriae (Figs. 1A, B; arrows). We also observed that individual, single cilia were displayed on the surface of secretory cells (Figs. 1A, B; arrowheads). Next, we processed transverse sections of the human FT at the level of infundibulum, ampulla, and isthmus (Fig. 2A). We stained for acetylated α-tubulin (a marker of ciliary axonemes in both MCCs and primary cilia) and γ-tubulin (a marker of the ciliary basal body). This revealed that single, primary cilia located on secretory cells had regional specification in the FT, being less abundant toward the infundibulum and more prolific toward the isthmus (Fig. 2B).

**FIGURE 3:**
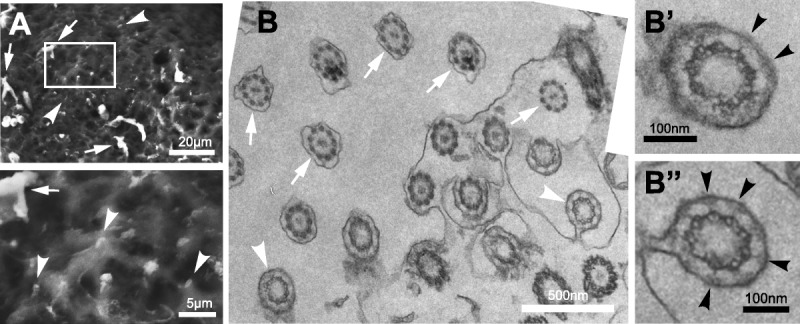
Scanning and transmission electron microscopy of cilia in the human FT. A, Scanning electron microscopy of fresh frozen mucosal layer tissue specimens of FT from the ampulla region. Arrows indicate MCCs and arrowheads indicate primary cilia, with detail shown in magnified insets. The frame indicates the region shown magnified in the lower panel. Scale bars 20 or 5 μm, as indicated. B, Transmission electron microscopy imaging of transverse sections of FT in the ampulla region, arranged in a photomosaic, at the level of transition zones in primary cilia. Arrows indicate MCCs and arrowheads indicate primary cilia. Scale bar = 500 nm. Magnified regions (B’ and B”) show individual primary cilia, with the Y-shaped links of the ciliary transition zone indicated by black arrowheads Scale bars = 100 nm.

Scanning electron microscopy of fresh processed FT tissue from the ampulla region also showed the presence of single cilia on the epithelial cell surface that could be readily discriminated from MCCs (Fig. 3A). Transmission electron microscopy of transverse sections enabled MCCs with a “9 + 2” microtubule ultrastructure (Fig. 3B; arrows) to be discriminated from occasional “9 + 0” primary cilia (Fig. 3B; white arrowheads). Primary cilia could also be discriminated by the presence of Y-shaped links (Figs. 3B’, B”; black arrowheads), which are a typical ultrastructural feature of the ciliary transition zone, and by the absence of either a basal plate or a central microtubule pair^33^,^34^ at this sectioning level (Fig. 3B). Because MCC originate from a single primary cilium,^3^–^5^,^35^ cells contain either a primary cilium or MCC but their distribution is mutually exclusive and the 2 cilia types do not occur in the same cells.

### Functional Characterization of Primary Cilia in the Human FT

**FIGURE 4:**
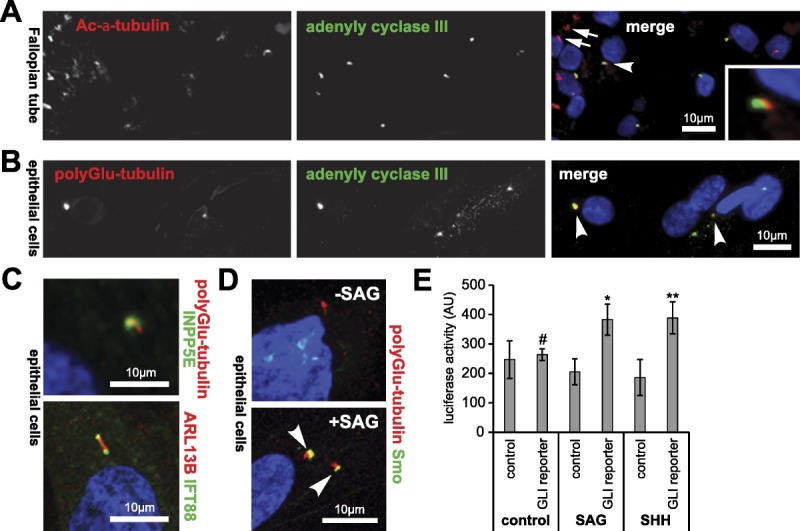
Functional characterization of primary cilia in the normal human FT and ex vivo cultured primary epithelial cells. A, IF staining of primary cilia in the mucosal layer of the ampullary region for acetylated (Ac) α-tubulin (red) and adenylate cyclase III (green). Representative example indicated by arrowhead and shown in detail in the magnified inset. The arrowhead indicates MCCs. Scale bar = 10 μm. B, Ex vivo cultured normal FT primary epithelial cells stained for polyglutamylated tubulin (red) and adenylate cyclase III (green), with representative primary cilia indicated by arrowheads. Scale bar = 10 μm. C, Cultured FT epithelial cells IF stained for the following markers of primary cilia function: upper panel, polyglutamylated tubulin (red), and INPP5E (green); lower panel, ARL13B (red) and IFT88 (green). Scale bars = 10 μm. D, Cultured FT epithelial cells IF stained for Smoothened (Smo; green) showing ciliary translocation (arrowheads) after 4 hours treatment with 2 μM SAG. Scale bar = 10 μm. E, Bar graph of Shh signaling activity (arbitrary units; AU) measured by the GLI luciferase reporter assay in isolated ex vivo FT epithelilal cells. Cells were transfected with either the GLI reporter construct or an inactive negative control construct followed by treatment with vehicle negative control, 1 μg/mL recombinant human Shh protein or 2 μM SAG. Statistical significance of pairwise comparisons between the GLI reporter and control constructs is indicated: #not significant, *P < 0.05, **P < 0.01 (paired Student *t* test for 3 biological replicates; error bars indicate s.e.m.).

To investigate the possible functional roles of primary cilia in the FT, we used IF staining for specific ciliary markers. Primary cilia, visualized by staining for acetylated α-tubulin, were approximately 2 μm in length (Fig. 4A). IF staining for the signaling intermediate adenylate cyclase III (ACIII) showed significant accumulation at the base of primary cilia in transverse tissue sections from the ampulla region (Fig. 4A; arrowhead), suggesting that these cilia were active in mediating ciliary signaling functions. ACIII appeared to be absent from MCCs (Fig. 4A; arrow). We then isolated primary epithelial cells from the mucosal layer of normal FT tissue for ex vivo culture. Immunofluorescence staining for polyglutamylated tubulin visualized primary cilia in cultured epithelial cells, with ACIII predominantly localized at the base of cilia (Fig. 4B). Fallopian tube epithelial cell cilia also carried other ciliary markers (INPP5E, ARL13B, and IFT88; Fig. 4C), suggesting that they had active signaling and ciliary transport functions. INPP5E is a ciliary phosphoinositide 5-phosphatase that regulates Hedgehog signaling.^34^,^36^ ARL13B is a member of the ARF family of small GTPases that regulates ciliary membrane formation.^37^ IFT88 is a component of the IFT-B complex of proteins that mediate both anterograde and retrograde intraflagellar transport of cargo proteins along the ciliary axoneme.^38^ IFT88 accumulated at both the ciliary base and tip (Fig. 4C), indicating that epithelial cell cilia had active transport processes.

To test this hypothesis, we assayed Shh signaling activity in ex vivo culture by treatment with low concentrations of the Smo agonist SAG. This induced translocation of the Shh signaling intermediate Smo into primary cilia (Fig. 4D), indicating that the cilia were responsive to Shh signaling and therefore functionally active in mediating normal ciliary signaling processes. Luciferase reporter assays, using a reporter construct containing GLI response elements, showed that specific ciliary Shh signaling was activated by treatment with both SAG and recombinant SHH protein (Fig. 4E). This indicated that FT secretory cells display primary cilia that are both functional and responsive to ciliary-mediated stimuli.

### Primary Cilia Are Absent in Serous Tubal Intraepithelial Carcinomas

**FIGURE 5:**
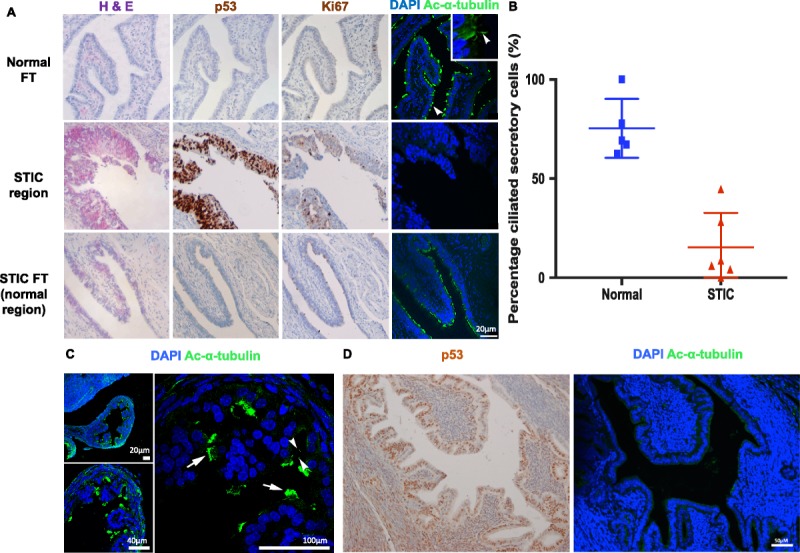
Loss of primary cilia in human and mouse STIC FT secretory cells. A, Images of serial longitudinal sections of paraffin-embedded human FT stained with H&E, anti-p53, and anti–Ki-67 antibodies. H&E staining was used to identify regions of STIC with confirmatory p53 and Ki-67 staining. Confocal images were captured at 40× magnification. Sections were stained with DAPI (blue) and anti–acetylated α-tubulin (green) to mark nuclei and ciliary axonemes, respectively. An example of a primary cilium is indicated by the arrowhead. Loss of epithelial cilia was observed in STIC regions identified by H&E on serial sections. Non-STIC epithelial regions within the same FT or matched FT still had an abundance of primary cilia. Scale bar = 20 μM. B, The incidence of primary cilia on secretory cells was quantified in the outermost layer of epithelial cells. The graph shows the percentage of secretory cells with cilia, calculated within each STIC region for patients 1 to 6, and in matched normal fimbrial end regions in either the same FT or the contralateral FT. Pairwise statistical comparison between the percentage of primary cilia on secretory cells in these regions was determined using an unpaired Student *t* test (*P* < 0.0002). One to 4 fields of view were used to determine the mean values for each sample; error bars indicate s.e.m. C, Wild-type mouse FT fimbriae sections stained for acetylated α-tubulin (green) to mark cilia were imaged at 20×, 40×, and 100× magnification. Nuclei were counterstained with DAPI (blue). Primary cilia are indicated by arrowheads and MCCs by arrows. D, The mouse STIC model shows loss of primary cilia from secretory cells. Left panel: regions of STIC were defined by H&E and p53 positive staining in the FT epithelium using IHC. Right panel: IF imaging at 40× magnification of the corresponding region of STIC, stained with DAPI (blue) and acetylated α-tubulin (green). Scale bars = 50 μm.

Since primary cilia are formed during the G_1_/G_0_ phases of the cell cycle, we reasoned that primary cilia would be absent in rapidly proliferating neoplastic tissue in the FT. We therefore investigated STIC, a precursor lesion of the FT that is recognized to be the source of most ovarian HGSC.^22^–^24^ As part of routine clinical assessment of 6 ovarian cancer patients, longitudinal sections of FFPE FT tissue samples were stained with H&E to identify STIC regions, which were subsequently confirmed by IHC to show overexpression of p53^39^ and increased nuclear levels of Ki-67 (a standard marker of cell proliferation)^40^ (Fig. 5A). In serial tissue sections, we also used IHC staining to assess the number of primary cilia in STIC lesions. A loss of primary cilia was observed in STIC regions, but non-STIC epithelial cells within the same FT (and/or similar regions in the contralateral normal FT) still had an abundance of primary cilia, suggesting that primary cilia loss was specific to STIC (Fig. 5A). Analysis of 6 samples revealed that STIC lesions had a statistically significant reduction in the average percentage of secretory cells with primary cilia compared with normal fimbrial tissue (*P* < 0.0002, Student *t* test) (Fig. 5B and Supplementary Table 1, http://links.lww.com/IGC/A798). We also investigated a mouse model of human FT STIC. Hematoxylin and eosin and p53-positive staining in the mouse FT defined regions of STIC. Primary cilia incidence on secretory cells was decreased in the mouse STIC in comparison to wild-type FT samples (Figs. 5C, D), confirming the result from human STIC samples.

### IHC Markers to Distinguish Between MCC and Primary Cilia

**FIGURE 6:**
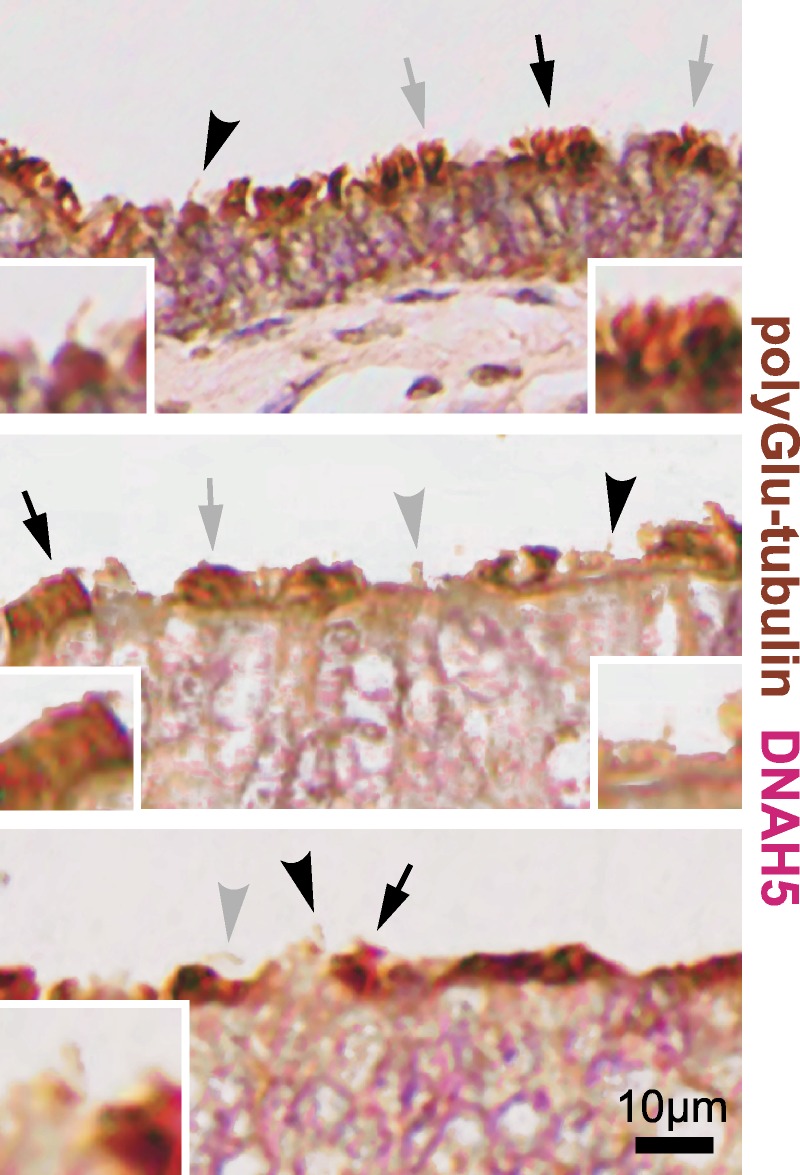
Dual IHC staining of normal human FT at the fimbrial end. The axonemes of MCC and primary cilia are visualized by staining for polyglutamylated tubulin (brown), and MCC were also visualized by staining for DNAH5 (red). Sections were counterstained with hematoxylin. Arrows indicate MCCs, and arrowheads indicate single immotile primary cilia on secretory peg cells. Black symbols indicate MCCs and primary cilia shown in detail for magnified insets. Scale bars = 10 μm.

To distinguish between MCC and primary cilia by conventional dual IHC staining, we stained a longitudinal section of the fimbrial end of a normal human FT for polyglutamylated tubulin, which identifies ciliary axonemes in both MCCs and primary cilia. We also stained for DYNE5, which is a marker of outer dynein arms in motile cilia.^41^ Multiple motile cilia were predominantly distributed at the apex of mucosal folds in the fimbriae (Fig. 6; arrows). However, single primary cilia were displayed on the surface of secretory cells (Fig. 6; arrowheads) and could be readily discriminated from MCC on ciliated cells by both morphology and IHC staining.

## DISCUSSION

Many recent studies have described the sensory roles of primary cilia. However, most previous studies of the FT have only described the presence of MCC that are not thought to have any direct sensory role.^27^,^28^ There are very few descriptions of primary cilia in the mammalian oviduct,^29^,^30^ and there have been no previous investigations of the possible functional roles of primary cilia in the FT. Here, we report the characterization of primary cilia displayed on secretory cells of the mucosal layer in the normal human FT. We show that primary cilia are remarkably abundant on secretory cells in the ampulla, isthmus, and, in particular, the fimbriae of the human FT. Furthermore, these cilia appeared to be fully responsive to ciliary-mediated stimuli and fully functional in mediating ciliary-mediated signaling pathways.

We suggest that, by analogy with other epithelial tissues such as renal tubules, primary cilia could detect paracrine signals in tubular fluid and mediate cellular homeostasis of secretory cells within the FT. Primary cilia have been suggested to be hubs for receiving extracellular signals and integrating intracellular signaling cascades,^3^,^4^,^7^ but the receptors and ligands that could mediate these processes in the FT epithelium are unknown. Further research could provide significant insights into the normal function of secretory cells, the regulation of sperm capacitation, and possible pathogenic mechanisms of idiopathic infertility.

To investigate the possible role of FT primary cilia in a relevant pathogenic process, we assessed the presence of primary cilia on secretory cells in STIC lesions. Recent advances in understanding the pathogenesis of ovarian cancer suggest that many HGSC originate from the FT fimbriae, and that STIC represents the putative precursor of these neoplasms.^22^–^24^ Evidence for this hypothesis is the observation that nearly all STIC contain p53 mutations similar to HGSC.^23^ We observed that primary cilia incidence was decreased on secretory cells in STIC lesions in both humans and a mouse model, but abundant in surrounding normal mucosal tissue. This finding complements an earlier study that identified primary cilia in normal ovarian surface epithelial cells and a reduction in ovarian cancers.^21^ We suggest that loss of primary cilia on secretory cells in the FT may be one of the early molecular events in benign mucosa that are associated with the transformation of tubal intraepithelial carcinoma into metastatic serous cancer. Further studies should be performed to assess if costaining for DYNE5 and polyglutamylated tubulin are useful IHC markers that provide discriminative value for specific primary cilia loss in early FT lesions, such as p53 signatures and serous tubal intraepithelial lesions. Similarly, STIC mimics such as endometrioid metaplasia and areas of reactive changes should be assessed to see if primary cilia are retained in these tissues to determine if primary ciliary loss is indeed an early event in ovarian cancer development.

Detailed characterization of the FT, the site of origin of ovarian HGSC, and its precursor lesion STIC are essential for the development of early detection markers and improved imaging screening techniques. Currently, STIC are diagnosed by H&E examination supported by Ki-67 and p53 IHC. However, there are a number of caveats to these diagnostic criteria. First, the morphological changes are subtle, which require considerable expertise to detect. Second, many STICs exhibit only a marginal elevation in Ki-67 proliferation index (**>**10% positive nuclei), and for a few samples, a p53 null mutation precludes the detection of p53 by IHC.^40^

To improve diagnostic rates, there is therefore a clinical need to identify new STIC biomarkers that could identify diagnostically challenging STIC samples. We propose that dual IHC staining for the polyglutamylated tubulin and DYNE5 ciliary markers should be assessed for clinical utility as a biomarker of STIC and early FT lesions. This has implications for the early detection, prevention, and the development of new therapeutic approaches to reduce mortality from ovarian cancer. The characterization of primary cilia on secretory cells in the FT is therefore important for a more complete understanding of the molecular pathogenesis of ovarian cancer and warrants further research.

## Supplementary Material

SUPPLEMENTARY MATERIAL

## References

[1] Ferenczy A Richart RM Agate FJJ Scanning electron microscopy of the human fallopian tube. *Science*. 1972;175:783–784. 5057820 10.1126/science.175.4023.783

[2] Patek E The epithelium of the human fallopian tube. A surface ultrastructural and cytochemical study. *Acta Obstet Gynecol Scand Suppl*. 1974;31:1–28. 4527061

[3] Berbari NF O’Connor AK Haycraft CJ The primary cilium as a complex signaling center. *Curr Biol*. 2009;19:R526–R535. 19602418 10.1016/j.cub.2009.05.025 PMC2814769

[4] Lancaster MA Gleeson JG The primary cilium as a cellular signaling center: lessons from disease. *Curr Opin Genet Dev*. 2009;19:220–229. 19477114 10.1016/j.gde.2009.04.008 PMC2953615

[5] Malicki J Johnson CA The cilium: cellular antenna and central processing unit. *Trends Cell Biol*. 2017;27:126–140. 27634431 10.1016/j.tcb.2016.08.002 PMC5278183

[6] Huangfu D Liu A Rakeman AS Hedgehog signalling in the mouse requires intraflagellar transport proteins. *Nature*. 2003;426:83–87. 14603322 10.1038/nature02061

[7] Goetz SC Anderson KV The primary cilium: a signalling centre during vertebrate development. *Nat Rev Genet*. 2010;11:331–344. 20395968 10.1038/nrg2774 PMC3121168

[8] Angers S Moon RT Proximal events in Wnt signal transduction. *Nat Rev Mol Cell Biol*. 2009;10:468–477. 19536106 10.1038/nrm2717

[9] Ezratty EJ Stokes N Chai S A role for the primary cilium in Notch signaling and epidermal differentiation during skin development. *Cell*. 2011;145:1129–1141. 21703454 10.1016/j.cell.2011.05.030 PMC3135909

[10] Corbit KC Aanstad P Singla V Vertebrate Smoothened functions at the primary cilium. *Nature*. 2005;437:1018–1021. 16136078 10.1038/nature04117

[11] Lancaster MA Schroth J Gleeson JG Subcellular spatial regulation of canonical Wnt signalling at the primary cilium. *Nat Cell Biol*. 2011;13:700–707. 21602792 10.1038/ncb2259 PMC3107376

[12] Boehlke C Kotsis F Patel V Primary cilia regulate mTORC1 activity and cell size through Lkb1. *Nat Cell Biol*. 2010;12:1115–1122. 20972424 10.1038/ncb2117 PMC3390256

[13] Bell PD Fitzgibbon W Sas K Loss of primary cilia upregulates renal hypertrophic signaling and promotes cystogenesis. *J Am Soc Nephrol*. 2011;22:839–848. 21493775 10.1681/ASN.2010050526 PMC3083306

[14] Habbig S Bartram MP Müller RU NPHP4, a cilia-associated protein, negatively regulates the Hippo pathway. *J Cell Biol*. 2011;193:633–642. 21555462 10.1083/jcb.201009069 PMC3166863

[15] Frank V Habbig S Bartram MP Mutations in NEK8 link multiple organ dysplasia with altered Hippo signalling and increased c-MYC expression. *Hum Mol Genet*. 2013;22:2177–2185. 23418306 10.1093/hmg/ddt070

[16] Clement CA Ajbro KD Koefoed K TGF-beta signaling is associated with endocytosis at the pocket region of the primary cilium. *Cell Rep*. 2013;3:1806–1814. 23746451 10.1016/j.celrep.2013.05.020

[17] Yuan K Frolova N Xie Y Primary cilia are decreased in breast cancer: analysis of a collection of human breast cancer cell lines and tissues. *J Histochem Cytochem*. 2010;58:857–870. 10.1369/jhc.2010.955856 PMC2942739 20530462

[18] Kim J Dabiri S Seeley ES Primary cilium depletion typifies cutaneous melanoma in situ and malignant melanoma. *PLoS One*. 2011;6:e27410. 22096570 10.1371/journal.pone.0027410 PMC3214062

[19] Seeley ES Carriere C Goetze T Pancreatic cancer and precursor pancreatic intraepithelial neoplasia lesions are devoid of primary cilia. *Cancer Res*. 2009;69:422–430. 19147554 10.1158/0008-5472.CAN-08-1290 PMC2629528

[20] Hassounah NB Nagle R Saboda K Primary cilia are lost in preinvasive and invasive prostate cancer. *PLoS One*. 2013;8:e68521. 23844214 10.1371/journal.pone.0068521 PMC3699526

[21] Egeberg D Lethan M Manguso R Primary cilia and aberrant cell signaling in epithelial ovarian cancer. *Cilia*. 2012;1:15. 23351307 10.1186/2046-2530-1-15 PMC3555760

[22] Crum CP Drapkin R Kindelberger D Lessons from BRCA: the tubal fimbria emerges as an original for pelvic serous cancer. *Clin Med Res*. 2007;5:35–44. 17456833 10.3121/cmr.2007.702 PMC1855333

[23] Kindelberger DW Lee Y Miron A Intraepithelial carcinoma of the fimbria and pelvic serous carcinoma: Evidence for a causal relationship. *Am J Surg Pathol*. 2007;31:161–169. 17255760 10.1097/01.pas.0000213335.40358.47

[24] Crum CP Drapkin R Miron A The distal fallopian tube: a new model for pelvic serous carcinogenesis. *Curr Opin Obstet Gynecol*. 2007;19:3–9. 17218844 10.1097/GCO.0b013e328011a21f

[25] Miyoshi I Takahashi K Kon Y Mouse transgenic for murine oviduct-specific glycoprotein promoter-driven simian virus 40 large T-antigen: tumor formation and its hormonal regulation. *Mol Reprod Dev*. 2002;63:168–176. 12203826 10.1002/mrd.10175

[26] Sherman-Baust CA Kuhn E Valle BL A genetically engineered ovarian cancer mouse model based on fallopian tube transformation mimics human high-grade serous carcinoma development. *J Pathol*. 2014;233:228–237. 24652535 10.1002/path.4353 PMC4149901

[27] Hagiwara H Harada S Maeda S Ultrastructural and immunohistochemical study of the basal apparatus of solitary cilia in the human oviduct epithelium. *J Anat*. 2002;200:89–96. 11833657 10.1046/j.0021-8782.2001.00004.x PMC1570880

[28] Hagiwara H Ohwada N Aoki T The primary cilia of secretory cells in the human oviduct mucosa. *Med Mol Morphol*. 2008;41:193–198. 19107608 10.1007/s00795-008-0421-z

[29] Crow J Amso NN Lewin J Morphology and ultrastructure of fallopian tube epithelium at different stages of the menstrual cycle and menopause. *Hum Reprod*. 1994;9:2224–2233. 7714136 10.1093/oxfordjournals.humrep.a138428

[30] Raidt J Werner C Menchen T Ciliary function and motor protein composition of human fallopian tubes. *Hum Reprod*. 2015;30:2871–2880. 26373788 10.1093/humrep/dev227

[31] Singh N Gilks CB Wilkinson N Assignment of primary site in high-grade serous tubal, ovarian and peritoneal carcinoma: a proposal. *Histopathology*. 2014;65:149–154. 24660659 10.1111/his.12419

[32] Dawe HR Smith UM Cullinane AR The Meckel-Gruber Syndrome proteins MKS1 and meckelin interact and are required for primary cilium formation. *Hum Mol Genet*. 2007;16:173–186. 17185389 10.1093/hmg/ddl459

[33] Szymanska K Johnson CA The transition zone: an essential functional compartment of cilia. *Cilia*. 2012;1:10. 23352055 10.1186/2046-2530-1-10 PMC3555838

[34] Fisch C Dupuis-Williams P Ultrastructure of cilia and flagella—back to the future! *Biol Cell*. 2011;103:249–270. 21728999 10.1042/BC20100139

[35] Chávez M Ena S Van Sande J Modulation of ciliary phosphoinositide content regulates trafficking and Sonic Hedgehog signaling output. *Dev Cell*. 2015;34:338–350. 26190144 10.1016/j.devcel.2015.06.016

[36] Garcia-Gonzalo FR Phua SC Roberson EC Phosphoinositides regulate ciliary protein trafficking to modulate Hedgehog signaling. *Dev Cell*. 2015;34:400–409. 26305592 10.1016/j.devcel.2015.08.001 PMC4557815

[37] Lu H Toh MT Narasimhan V A function for the Joubert syndrome protein Arl13b in ciliary membrane extension and ciliary length regulation. *Dev Biol*. 2015;397:225–236. 25448689 10.1016/j.ydbio.2014.11.009

[38] Follit JA Xu F Keady BT Characterization of mouse IFT complex B. *Cell Motil Cytoskeleton*. 2009;66:457–468. 19253336 10.1002/cm.20346 PMC2753169

[39] Cass I Walts AE Barbuto D A cautious view of putative precursors of serous carcinomas in the fallopian tubes of BRCA mutation carriers. *Gynecol Oncol*. 2014;134:492–497. 25026639 10.1016/j.ygyno.2014.07.084

[40] Kuhn E Kurman RJ Sehdev AS Ki-67 labeling index as an adjunct in the diagnosis of serous tubal intraepithelial carcinoma. *Int J Gynecol Pathol*. 2012;31:416–422. 22833080 10.1097/PGP.0b013e31824cbeb4 PMC3715095

[41] Olbrich H Haffner K Kispert A Mutations in DNAH5 cause primary ciliary dyskinesia and randomization of left-right asymmetry. *Nature Genet*. 2002;30:143–144. 11788826 10.1038/ng817

