# Supplementary material for: Characterization of Primary Cilia in Normal Fallopian Tube Epithelium and Serous Tubal Intraepithelial Carcinoma
Source: Int J Gynecol Cancer. 2018 Aug 8;28(8):1535–44. doi: 10.1097/IGC.0000000000001321 (PMC6166701; doi:10.1097/IGC.0000000000001321)
Supplement: SUPPLEMENTARY MATERIAL [file igj-28-1535-s001.docx]

**Supplementary Table 1. Percentage of STIC secretory cells with cilia**

| **Patient No.** | **Sample Type** | **Percentage of secretory cells with cilia +/- SE (fields of view)** | |
| --- | --- | --- | --- |
|  |  | **Normal** | **STIC (Ki67 & p53 +ve)** |
| 1 | RFT STIC | 69.2 +/- 3 (3) | 0 (3) |
|  | LFT Normal | 77.84 +/- 5 (2) |  |
| 2 | LFT STIC | 67.18 +/- 19.9 (4) | 4.17 +/- 7.2 (3) |
| 3 | RFT STIC |  | 8.7 (1) |
| 4 | LFT STIC | 62.5 +/- 31 (2) | 6 +/- 5.88 (3) |
| 5 | LFT STIC |  | 44.66 +/- 9.9 (3) |
| 6 | RFT STIC | 100 (1) | 28.21 +/- 7.2 (2) |

RFT; right fallopian tube, LFT; left fallopian tube; STIC; serous tubal intraepithelial carcinoma
